# Surgical Shock*Impromptu remarks before the Medical Association of Georgia, April, 1894.

**Published:** 1894-06

**Authors:** Richard Douglas

**Affiliations:** Nashville, Tenn.


### Southern Medical Record.

A MONTHLY JOURNAL OF MEDICINE AND SURGERY.

VOL. XXIV. ATLANTA, GA., JUNE, 1894. No. 6.

### Original Articles.

### SURGICAL SHOCK.*

BY RICHARD DOUGLAS, M.D., NASHVILLE, TENN.

Dr. Westmoreland has courteously insisted that I should occupy the time allotted to him with some remarks upon any subject of my selection. I regret his great hospitality deprives the Association of the pleasure of hearing his interesting paper, and, that it shall not entirely go by default, I shall in a few imperfect remarks address myself to his subject, which he will, of course, elaborate.

Surgical shock is the subject of the greatest possible interest to us all. It is not one of those subjects that unfortunately is brought into the proceedings of a general body like this, and is only of interest to a few. The specialist sometimes, in his presumption and egotism (I can say this, for I happen to be that sort of a fellow myself), assumes that the general audience is interested in his particular fad or theme. This paper, however, of Dr. Westmoreland's commands the interest of all. Shock, as the surgeon meets with it and considers it, is due to excessive or overstimulation of the sensory nerves. There are so many factors as causal elements, it is often difficult to designate the special condition or

*Impromptu remarks before the Medical Association of Georgia, April, 1894.

circumstance giving rise to it. The injury itself to the vital tissues, the loss of blood, the circumstances attending surgical operation and mental impressions, all enter as causes. Profound shock, such as the surgeon is called upon to relieve, is a familiar picture to you all. You at once recognize it in the compressible and intermittent pulse, the hurried thoracic respiration or the deep sigh, the relaxed condition of the general muscular system, the profuse diaphoresis, the pinched countenance, the collapse of the curve of the lips, and the voluntary escape of urine and feces. It is important that we should differentiate this condition from concealed hemorrhage, and in surgery of the abdomen especially should we ever bear in mind that the so-called secondary shock is in reality but a symptom of internal hemorrhage due to imperfect surgery. Memory of one of my early cases haunts me still. The surgeon is sometimes called upon to relieve shock, but more frequently to prevent it. I was much impressed with the words of our eloquent orator, Dr. Benedict, when he said this morning there was much in mental impression. I believe there is much in impressing the patient with confidence in you. So deport yourself that the patient may feel that his trust is well founded. If the patient is thoroughly imbued with the idea that the operation is the last of him, and that the surgeon has small hopes of his recovery, such patient is sure to sustain the full measure of shock. The manner of administering the anesthetic greatly influences shock. I think it wise to precede the administration of ether with the hypodermatic injection of morphia and atropia. I am one of those who believe in the minimum amount of ether that is necessary. I am fully aware that some surgeons use half a pound to a pound of ether during an ordinary operation, completely waterlogging their patient with it. I carry the patient under ether slowly, not forced nor strangulated nor drowned with it. The temperature of the operating room should be about 75°—a little uncomfortable, I grant you, for the surgeon, but better for the patient. With the introduction of the practice of aseptic surgery, it was quite the fashion for the surgeon to cover the patient with towels saturated with bichloride water. I know of no measure so deleterious to the patient—no step in the technique so certain to favor shock as this. You can accomplish all you wish with dry sterilized towels. The wet ones ruin the instruments, irritate the skin, and in cooling chill your patient. By the careful administration of the anesthetic, by securing the proper temperature for the operating room, by keeping the patient dry and warm, with the extremities well wrapt in blankets, I think we may in a great measure prevent shock. Whether the operation be one in general or special surgery, it should be expeditiously performed. While I deprecate hurried and incomplete methods, I think the habit of many surgeons, who stand and leisurely wash their hands to demonstrate the technique to students, is to be strongly condemned. The patient should receive absolute and undivided attention of the operator. "Chronic surgery"—that is, unnecessarily prolonged operations—is responsible oftentimes for shock. Unavoidable hemorrhage may be the cause of shock; if so we should take means to restore the patient at once. Sufficient attention has not been given to intra-arterial injections to overcome the acute anemic at the time of operation. This is particularly valuable in railroad surgery. The three most important agents that we possess to overcome shock are, first, if due to hemorrhage, injections of saline solutions directly into femoral artery; high rectal injections of hot saline solutions; if due simply to sensory disturbance, strychnine, digitalis and atropia are our remedies.

In regard to the propriety of operating when the patient is under shock, I do not know that this phase of the subject is within the province of this discussion. Railway surgeons have to contend with that more than any of us. It is the habit of some gentlemen to lop off limbs as fast as they can get them in order to secure a better fee. I know, gentlemen, that this is so, and the practice should be deprecated. In the cases of mutilation of the limb, it appears to me it is altogether unnecessary to do anything except to provide against the occurrence of hemorrhage, protect the limb and endeavor to prevent decomposition and infection and wait for reaction. If the patient does not react, he would have died anyway, and surgery would have received the opprobrium.

One word more and I have done. In abdominal operations there is one condition that often occurs, namely: a condition of profound nausea attended by shock. In that condition I have employed with the most satisfactory results two measures, namely: high rectal injection and irrigation of the stomach. In such cases I have washed out the stomach and resorted to rectal injections, and have always found that this method is productive of more good and quicker stimulation than any other.

I thank the Association for the courtesy it has extended to me, and wish to say this: standing here as I do, at the feet of the Nestor of Southern surgery—a man whose work has made for him and your State an everlasting name (I refer to Dr. Gaston), I think it is the height of presumption that I should further trespass upon your time.

